# An explicit unconditionally stable scheme: application to diffusive Covid-19 epidemic model

**DOI:** 10.1186/s13662-021-03513-7

**Published:** 2021-08-03

**Authors:** Yasir Nawaz, Muhammad Shoaib Arif, Kamaleldin Abodayeh, Wasfi Shatanawi

**Affiliations:** 1 Department of Mathematics, Air University, PAF Complex E-9, Islamabad, 44000 Pakistan; 2 Department of Mathematics and General Sciences, Prince Sultan University, Riyadh, Saudi Arabia; 3 Department of Medical Research, China Medical University Hospital, China Medical University, Taichung, 40402 Taiwan; 4 Department of Mathematics, Hashemite University, Zarqa, Jordan

**Keywords:** Proposed scheme, Conditionally positivity preserving, Diffusive COVID-19 model, Stability, Convergence conditions

## Abstract

An explicit unconditionally stable scheme is proposed for solving time-dependent partial differential equations. The application of the proposed scheme is given to solve the COVID-19 epidemic model. This scheme is first-order accurate in time and second-order accurate in space and provides the conditions to get a positive solution for the considered type of epidemic model. Furthermore, the scheme’s stability for the general type of parabolic equation with source term is proved by employing von Neumann stability analysis. Furthermore, the consistency of the scheme is verified for the category of susceptible individuals. In addition to this, the convergence of the proposed scheme is discussed for the considered mathematical model.

## Introduction

Mathematical modeling of epidemic diseases is one of the branches of modeling concerned with somehow estimating and predicting some insight into actual disease. In the literature, the constructed mathematical models for epidemic diseases were the first-order differential equations system that might have been constructed on some assumptions. SIR models belong to the constructed mathematical models of epidemic diseases that can describe some relationships between susceptible, infected, and recovered individuals in COVID-19 epidemic disease. In [1] presented the SIR model that contained health medication factor, initial infected, transmits factor, and human contact factor. One of the concluded results was decreasing COVID-19 spreading by choosing a low contact factor and high medication factor. Reference [2] has consisted of the SEIR model of COVID-19 that contained isolation factors and vaccination as model parameters. The basic reproduction number is found by using the generation matrix method, and the global stability of the given model has also been discussed. The modification of the classical SIR model has been shown in [2] by proposing a susceptible-infected-removed-sick (SIRSi) computational model. The proposed model in [2] considered the level of immunity within the population and asymptomatic cases. The SEIR epidemic model given in [3] used a convex incidence rate. The simulations were obtained by applying the nonstandard finite difference method. An $S L_{1} L_{2} I_{1} I_{2} A_{1} A_{2} R$ epidemic model has been formulated in [4] for spreading an epidemic within the population. The model used an Erlang distribution of time of sojourn in considered compartments. In [5], a simple compartmental Kermack-McKendrick-type epidemic model was introduced with homogeneous and heterogeneously mixed populations. For the dynamics of COVID-19, a primer for analyzing, formulating, and simulating mathematical models were also given.

Some existing models can be used for the COVID-19 epidemic to see some insight into the epidemic disease. The mathematical model considered in [6] has consisted of susceptible, exposed, asymptomatic, infected, and recovered individuals. Exposed were those individuals that had pathogen but cannot transmit it to other individuals. At the same time, asymptomatic individuals could transmit the pathogen. However, they do not know about it and are infected. They knew that they had the disease and can transmit it since quarantine is another category of individuals considered in COID-19 epidemic disease. However, in the present modeling, quarantine individuals are not considered, although they can be regarded as infected individuals. Because if someone is infected, then it means that the individual knows about the disease. This infected individual can be considered one of the quarantine individuals, but quarantine can be regarded as the category of infected and under treatment people. So, for COVID-19 disease, infected and quarantine individuals can be considered to be the same. It is also assumed that the recovered individuals are not shifted to exposed or asymptomatic or infected individuals. The recovered individuals can be assumed to have an ignorable chance of being infected again. For the present modeling of COVID-19, it is also assumed that exposed people cannot be shifted into the category of recovered people.

Some of the numerical solutions for epidemic models included the diffusion effects that can be found in [7–9]. [10] has presented a predictor-corrector system to find a solution to large time values for obtaining insight into an epidemic to limit behavior. Variational iteration method and successive approximation methods have been applied in [11] to solve the SIR epidemic model with a constant vaccination strategy. The existing variational iteration method was shown to be inaccurate for the large domain. The existing variational iteration method was improved and identical to the successive approximation method. The modified method was more accurate than the existing one, given in [11]. The susceptible, exposed, infected, diagnosed, recovered (SEIJR) epidemic model was considered in [12] with effects of net inflow of people into a region. Different initial population distributions were considered with the considered model, and it was solved by the numerical method for analyzing the transmission of disease. A diffusive epidemic model [13] has been investigated for describing the transmission of influenza as an epidemic. The spread of the disease showed that diffusion and initial population distribution played an important role.

The COVID 19 pandemic is a worldwide destructive disease that raised severe health issues. It is considered one of the most devastating crises after World War II as it increased the death toll by 1,458,000, which is still rampant. This pandemic surged social issues and the economic recession and environmental disability that led to the destruction of habits, trade, economic relations, forms of work, and political organizations. Reportedly, this disease imparts curb on social movement more than 4 billion people.

Globally, all government and private healthcare departments were unprepared for this trauma which was simply a matter of time that arrived now. Pathogenic disorders wreak havoc in society in the past few months, for which mathematical modeling is the best way to investigate and control them once they enter the community. Nowadays, coronavirus is an essential topic for researchers as regards finding its treatment as the effectiveness and deaths are unbridled.

The virus was first reported in December 2019 in a Chinese city, Wuhan, as an infective agent named coronavirus [14, 15]. The viral disease COVID-19 is primarily transferred using droplets produced by infected persons. The infection is transmitted through droplets that are so dense than air particles and immediately fall on the ground, created due to sneezing and coughing of the infected person. COVID-19 confirmed cases reached 4 million earlier in more than 180 countries, and approximately 1,458,000 people have become victims of this dreadful virus [16].

A retrofitted state SIR system to task the overall number of sick circumstances and the specialized obligations on hemodialysis units and hospitals are presented [17]. Nesteruk observed the coronavirus epidemic trying to spread numbers based on assumptions throughout mainland China regrettably. The majority of casualties of COVID are predicted to become much higher than that forecast on February 2020; two days later, 12289 confirmed cases were added. Additional research focuses on updating predictions using up-to-date data and applying more convoluted mathematical representations. There does not exist any approved vaccine or diagnostic drugs for the avoidance and cure of coronavirus. However, research studies on potential antiviral drugs and vaccine candidates are under way in several countries. Vaccine evaluations, growth, and allocation are usually a big task than clinical trials. It is unlikely that the COVID-19 flu shot will be mentally prepared by 2021 within the shortest time possible. The dreadful germ can spread quickly in closely packed locations. Social detachment or low contact rate refers to increasing the disk environment among both people to delay infection spread [18–21]. They have studied the SIR model to guesstimate the adult location of the coronavirus infectious disease [22].

## Diffusive epidemic models

In the literature, some diffusive epidemic models have existed. From these, [23] investigated a diffusive model for the transmission of influenza as an epidemic. The equations have been tackled with the splitting method using different initial conditions of population density. Another diffusive epidemic model of $H1N1$ has been formulated [24] for gaining a basic understanding of virus behavior. It was assumed that all newborns were susceptible, and also, it was assumed that the mortality rate of individuals is greater than the natural mortality rate. Among the diffusive epidemic models, a vaccinated diffusive epidemic model has also been developed [25] for exploring the impact of diffusion and vaccination on the transmission of dynamics of influenza. In this work [25], a basic reproduction number was found for both vaccinated and non-vaccinated cases. Based on parameters in the system, sensitivity analysis of the reproduction number has been investigated. HIV/AIDS is incurable for human beings mentioned in [26], and a diffusive compartmental model of HIV/AIDS has been proposed with a delay process. The proposed scheme had the advantage of producing a positive solution. The stability and consistency have been given. A nonlinear model for Immunodeficiency Virus (*HIV*) has been proposed in [27]. For boundedness and wellposedness, theorems and propositions have been constructed, and the model was solved by employing the evolutionary Padé-approximation technique. This is some literature given on diffusive epidemic models. The reader can find more work on diffusive epidemic models by referring to [23–27]. In [28] an SEIR epidemic model for COVID-19 is constructed using several common control strategies, including hospitalization, quarantine, and external input. The particle swarm optimization (PSO) algorithm is used to estimate the system’s parameters using data from Hubei province.

In this contribution, a numerical scheme is proposed to solve the COVID-19 epidemic model with diffusion. The scheme is shown to be unconditionally stable for the considered type of epidemic models. The scheme is first-order accurate because it is constructed to provide the first-order accuracy in time and second-order accuracy in space for diffusion contained epidemic models. The scheme provides a conditionally positive solution. The conditions of finding positive solutions are found in the construction of the proposed scheme. The scheme is constructed on three-time levels, and it is an explicit scheme. The convergence of the modified epidemic model’s scheme is also discussed by applying the condition of convergence of infinite geometric series. Since the scheme is constructed on three-time levels, so it requires another scheme to be implemented at the first time level. The proposed scheme can be useful in those mathematical models where the positive solution is required to be found. Other than epidemic models, it can also be applied to solving any time-dependent partial differential equations which contain a diffusion term.

The model given in [6] is modified with diffusion effects, and the modified diffusive COVID-19 model is expressed as

1 $$\begin{aligned}& \frac{\partial S}{\partial t} = d_{1} \frac{\partial ^{2} S}{\partial x^{2}} - \alpha AS - \beta IS, \end{aligned}$$

2 $$\begin{aligned}& \frac{\partial E}{\partial t} = d_{2} \frac{\partial ^{2} E}{\partial x^{2}} + \alpha AS + \beta IS - \gamma E, \end{aligned}$$

3 $$\begin{aligned}& \frac{\partial A}{\partial t} = d_{3} \frac{\partial ^{2} A}{\partial x^{2}} + \gamma E - \sigma A - \mu A, \end{aligned}$$

4 $$\begin{aligned}& \frac{\partial I}{\partial t} = d_{4} \frac{\partial ^{2} I}{\partial x^{2}} + \sigma A - \mu I, \end{aligned}$$

5 $$\begin{aligned}& \frac{\partial R}{\partial t} = d_{5} \frac{\partial ^{2} R}{\partial x^{2}} + \mu A + \mu I. \end{aligned}$$

The boundary conditions corresponding to Eq. (1)–(5) are expressed as:

6 $$ \frac{\partial S}{\partial x} =0,\qquad \frac{\partial E}{\partial x} =0,\qquad \frac{\partial A}{\partial x} =0,\qquad \frac{\partial I}{\partial x} =0,\qquad \frac{\partial R}{\partial x} =0\quad \text{at } x =0, L $$

and the initial conditions are expressed as

7 $$ \begin{gathered} S ( x,0 ) = f_{1} ( x ),\qquad E ( x,0 ) = f_{2} ( x ), \qquad A ( x,0 ) = f_{3} ( x ),\\ I ( x,0 ) = f_{4} ( x ),\qquad R ( x,0 ) = f_{5} ( x ). \end{gathered} $$

Moreover, Table 1 provides a summary of the physical meaning of the parameters used in this model. For $d_{1} = d_{2} = d_{3} = d_{4} = d_{5} =0$, Eqs. (1)–(5) become ordinary differential equations, and linear stability of the system is determined by the Jacobean at the equilibrium point $( 1,0,0,0,0 )$ given in [1] as

8 $$ J = \begin{bmatrix} 0 & 0 & - \alpha & - \beta & 0\\ 0 & - \gamma & \alpha & \beta & 0\\ 0 & \gamma & - \beta - \mu & 0 & 0\\ 0 & 0 & \sigma & - \mu & 0\\ 0 & 0 & \mu & \mu & 0 \end{bmatrix}. $$

**Table 1:** Table of the physical meanings of the parameters used in the Diffusive COVID-19 Epidemic model investigated in this work

Symbols for variables	Description	Symbols for parameters	Description
*S*	Fraction of Susceptible	*α*	infecting rate of asymptomatic individuals
*E*	Fraction of Exposed	*β*	infecting rate of infectious individuals
*A*	Fraction of Asymptomatic	*γ*	a conversion rate of exposed individuals to become asymptomatic
*I*	Fraction of Infective	*σ*	rate of those fraction of infectious hosts which have symptoms
*R*	Fraction of Recovered	*μ*	the healing rate of asymptomatic and infectious individuals
		$d_{i} 's$ 1 ≤ *i* ≤ 5	Diffusion coefficients

In [1], two eigenvalues of the Jacobean (8) are zero, and the remaining eigenvalues can be found from the following polynomial:

$$ R ( \lambda ) = \beta \gamma \sigma - ( \mu + \lambda )\bigl[ ( \lambda + \mu ) ( \sigma + \mu + \lambda ) + \alpha \gamma \bigr]. $$

Using the Routh Hurwitz criteria for linear stability of the system (1)–(5), real parts of the eigenvalues must be negative, and this gives the condition for stability [1].

9 $$ \mu ( \mu + \sigma ) > \alpha \mu + \beta \gamma . $$

## Numerical scheme

The proposed numerical scheme can solve systems of Eqs. (1)–(5). At this stage, the construction of the numerical scheme is given. The scheme is constructed for Eq. (1) and the remaining Eqs. (2)–(5) will be discretized later on. Consider the following difference equation with unknown parameter *a*:

10 $$ \frac{S_{i}^{n +1} - S_{i}^{n - 1}}{\Delta t} = a \biggl\{ d_{1} \frac{S_{i +1}^{n} - 2 S_{i}^{n +1} + S_{i - 1}^{n}}{ ( \Delta x )^{2}} - \alpha A_{i}^{n} S_{i}^{n +1} - \beta I_{i}^{n} S_{i}^{n +1} \biggr\} . $$

We expand $S_{i}^{n +1}$ and $S_{i}^{n - 1}$ using Taylor series, as follows:

11 $$\begin{aligned}& S_{i}^{n +1} = S_{i}^{n} + \Delta t \biggl( \frac{\partial S}{\partial t} \biggr)_{i}^{n} + O \bigl( ( \Delta t )^{2} \bigr), \end{aligned}$$

12 $$\begin{aligned}& S_{i}^{n - 1} = S_{i}^{n} - \Delta t \biggl( \frac{\partial S}{\partial t} \biggr)_{i}^{n} + O \bigl( ( \Delta t )^{2} \bigr). \end{aligned}$$

Substituting the Taylor series expansions (11) and (12) into Eq. (10) yields

13 $$\begin{aligned}& S_{i}^{n} + \Delta t \biggl( \frac{\partial S}{\partial t} \biggr)_{i}^{n} + O \bigl( ( \Delta t )^{2} \bigr) \\& \quad = S_{i}^{n} - \Delta t \biggl( \frac{\partial S}{\partial t} \biggr)_{i}^{n} + O \bigl( ( \Delta t )^{2} \bigr)+ a \Delta t \biggl\{ d_{1} \frac{S_{i +1}^{n} - 2 S_{i}^{n +1} + S_{i - 1}^{n}}{ ( \Delta x )^{2}} - \frac{2 \Delta t}{ ( \Delta x )^{2}} \biggl( \frac{\partial S}{\partial t} \biggr)_{i}^{n} \\& \qquad {} - \alpha A_{i}^{n} S_{i}^{n} - \Delta t\alpha A_{i}^{n} \biggl( \frac{\partial S}{\partial t} \biggr)_{i}^{n}- \beta I_{i}^{n} S_{i}^{n} - \Delta t \beta I_{i}^{n} \biggl( \frac{\partial S}{\partial t} \biggr)_{i}^{n} + O \bigl( ( \Delta t )^{2} \bigr) \biggr\} . \end{aligned}$$

Equation (13) is further simplified as

14 $$\begin{aligned} 2 \Delta t \biggl( \frac{\partial S}{\partial t} \biggr)_{i}^{n} + O \bigl( ( \Delta t )^{2} \bigr) =& a \Delta t \biggl\{ \biggl( \frac{\partial S}{\partial t} \biggr)_{i}^{n} - \frac{2 \Delta t}{ ( \Delta x )^{2}} \biggl( \frac{\partial S}{\partial t} \biggr)_{i}^{n} \\ & {}- \Delta t\alpha A_{i}^{n} \biggl( \frac{\partial S}{\partial t} \biggr)_{i}^{n} - \Delta t\beta I_{i}^{n} \biggl( \frac{\partial S}{\partial t} \biggr)_{i}^{n} + O \bigl( ( \Delta t )^{2} \bigr) \biggr\} . \end{aligned}$$

Comparing coefficients of $\Delta t ( \frac{\partial S}{\partial t} )_{i}^{n}$ on both sides of Eq. (14) gives

15 $$ 2= a \biggl( 1 - \frac{2 \Delta t}{ ( \Delta x )^{2}} - \Delta t\alpha A_{i}^{n} - \Delta t\beta I_{i}^{n} \biggr). $$

Solving Eq. (15) gives the expression for *a*,

16 $$ a = \frac{2}{1 - \frac{2 \Delta t}{ ( \Delta x )^{2}} - \Delta t ( \alpha A_{i}^{n} - \beta I_{i}^{n} )}. $$

Thus Eqs. (1)–(5) are discretized as

17 $$\begin{aligned}& S_{i}^{n +1} = S_{i}^{n - 1} + \Delta t a_{1} \biggl\{ d_{1} \frac{S_{i +1}^{n} - 2 S_{i}^{n +1} + S_{i - 1}^{n}}{ ( \Delta x )^{2}} - \alpha A_{i}^{n} S_{i}^{n +1} - \beta I_{i}^{n} S_{i}^{n +1} \biggr\} , \end{aligned}$$

18 $$\begin{aligned}& E_{i}^{n +1} = E_{i}^{n - 1} + \Delta t a_{2} \biggl\{ d_{2} \frac{E_{i +1}^{n} - 2 E_{i}^{n +1} + E_{i - 1}^{n}}{ ( \Delta x )^{2}} + \alpha A_{i}^{n} S_{i}^{n} + \beta I_{i}^{n} S_{i}^{n} - \gamma E_{i}^{n +1} \biggr\} , \end{aligned}$$

19 $$\begin{aligned}& A_{i}^{n +1} = A_{i}^{n - 1} + \Delta t a_{3} \biggl\{ d_{3} \frac{A_{i +1}^{n} - 2 A_{i}^{n +1} + A_{i - 1}^{n}}{ ( \Delta x )^{2}} +\gamma E_{i}^{n} - \sigma A_{i}^{n+1} - \mu A_{i}^{n+1} \biggr\} , \end{aligned}$$

20 $$\begin{aligned}& I_{i}^{n +1} = I_{i}^{n - 1} + \Delta t a_{4} \biggl\{ d_{4} \frac{I_{i +1}^{n} - 2 I_{i}^{n +1} + I_{i - 1}^{n}}{ ( \Delta x )^{2}} + \sigma A_{i}^{n} - \mu I_{i}^{n +1} \biggr\} , \end{aligned}$$

21 $$\begin{aligned}& R_{i}^{n +1} = R_{i}^{n - 1} + \Delta t a_{5} \biggl\{ d_{4} \frac{R_{i +1}^{n} - 2 R_{i}^{n +1} + R_{i - 1}^{n}}{ ( \Delta x )^{2}} + \mu A_{i}^{n} + \mu I_{i}^{n} \biggr\} . \end{aligned}$$

The expressions for $a_{1}$, $a_{2}$, $a_{3}$, $a_{4}$ and $a_{5}$ are

22 $$ \begin{gathered} a_{1} = a_{1},\qquad a_{2} = \frac{2}{1 - 2 \frac{\Delta t}{ ( \Delta x )^{2}} - \Delta t\gamma },\qquad a_{3} = \frac{2}{1 - 2 \frac{\Delta t}{ ( \Delta x )^{2}} - \Delta t ( \sigma + \mu )},\\ a_{4} = \frac{2}{1 - 2 \frac{\Delta t}{ ( \Delta x )^{2}} - \Delta t\mu },\qquad a_{5} = \frac{2}{1 - 2 \frac{\Delta t}{ ( \Delta x )^{2}}}. \end{gathered} $$

Equations (17)–(21) are discretized equations, and the discretization is performed by employing the proposed scheme on Eqs. (1)–(5) and the unknowns $a_{i} $ $i =1,2, 3, 4, 5$ can be found from Eq. (22).

### Theorem

*The scheme produces a conditionally positive solution provided that positive initial conditions are chosen*, *and the solution is positive at the first time level*.

### Proof

Suppose $a= a_{1}, a_{2}, a_{3}, a_{4}, a_{5} \geq 0$. Then Eqs. (17)–(21) can be expressed as

22a $$\begin{aligned}& S_{i}^{n +1} = \frac{S_{i}^{n - 1} + \Delta t a_{1} \{ d_{1} \frac{S_{i +1}^{n} + S_{i - 1}^{n}}{ ( \Delta x )^{2}} \} }{1+2d d_{1} a_{1} + \alpha \Delta t a_{1} A_{i}^{n} +\beta \Delta t a_{1} I_{i}^{n}} \geq 0, \end{aligned}$$

22b $$\begin{aligned}& E_{i}^{n +1} = \frac{E_{i}^{n - 1} + \Delta t a_{2} \{ d_{2} \frac{E_{i +1}^{n} + E_{i - 1}^{n}}{ ( \Delta x )^{2}} + \alpha A_{i}^{n} S_{i}^{n} + \beta I_{i}^{n} S_{i}^{n} \} }{1+2d d_{2} a_{2} + \gamma \Delta t a_{2}} \geq 0, \end{aligned}$$

22c $$\begin{aligned}& A_{i}^{n +1} = \frac{A_{i}^{n - 1} + \Delta t a_{3} \{ d_{3} \frac{A_{i +1}^{n} + A_{i - 1}^{n}}{ ( \Delta x )^{2}} +\gamma E_{i}^{n} \} }{1+2d d_{3} a_{3} + \Delta t a_{3} ( \sigma + \mu )} \geq 0, \end{aligned}$$

22d $$\begin{aligned}& I_{i}^{n +1} = \frac{I_{i}^{n - 1} + \Delta t a_{4} \{ d_{4} \frac{I_{i +1}^{n} + I_{i - 1}^{n}}{ ( \Delta x )^{2}} + \sigma A_{i}^{n} \} }{1+2d d_{4} a_{4} + \Delta t \mu a_{4}} \geq 0, \end{aligned}$$

22e $$\begin{aligned}& R_{i}^{n +1} = \frac{R_{i}^{n - 1} + \Delta t a_{5} \{ d_{4} \frac{R_{i +1}^{n} + R_{i - 1}^{n}}{ ( \Delta x )^{2}} + \mu A_{i}^{n} + \mu I_{i}^{n} \} }{1+2d d_{5} a_{5}} \geq 0. \end{aligned}$$

So, the explicit relationships (22a)–(22e) show that the scheme will produce a positive solution at each time level with the first-order accuracy in time and second-order accuracy in space. The two positive initial conditions can be provided to apply the proposed scheme instead of employing any other scheme on the first time level, which will be constructed on two time levels. □

## Stability analysis

The stability of the proposed scheme is checked by employing the von Neumann stability criteria. Before starting the procedure of von Neumann stability criteria, consider the general form of the epidemic model given by

23 $$ \frac{\partial u}{\partial t} = \alpha _{1} \frac{\partial ^{2} u}{\partial x^{2}} - \beta _{1} u, $$

where *u* can be considered as one of the susceptible, exposed, infectious, asymptomatic, or recovered individuals and $\alpha _{1}$, $\beta _{1}$ are some rates.

Employing the proposed scheme in Eq. (23) yields

24 $$ u_{i}^{n +1} - = \overline{a} \biggl( \alpha _{1} \frac{u_{i +1}^{n} - 2 u_{i}^{n +1} + u_{i - 1}^{n}}{ ( \Delta x )^{2}} - \beta _{1} u_{i}^{n +1} \biggr), $$

where $\overline{a} = \frac{2}{1 - 2 d - \Delta t \beta _{1}}$.

By following the von Neumann stability criteria, the dependent components in the scheme (24) are expressed as

25 $$ u_{i \pm 1}^{n} = U^{n} e^{ ( i \pm 1 ) \overline{I} \theta },\qquad u_{i}^{n \pm 1} = U^{n \pm 1} e^{i \overline{I} \theta }, $$

where $\overline{I} = \sqrt{- 1}$. Applying transformation (25) to Eq. (24), one obtains

26 $$\begin{aligned} U^{n +1} e^{i \overline{I} \theta } - U^{n - 1} e^{i \overline{I} \theta } =& \Delta t \overline{a} \biggl( \alpha _{1} \biggl( \frac{e^{ ( i +1 ) \overline{I} \theta } + e^{ ( i - 1 ) \overline{I} \theta }}{ ( \Delta x )^{2}} \biggr) U^{n} \\ &{}- \frac{2 \alpha _{1}}{ ( \Delta x )^{2}} e^{i \overline{I} \theta } U^{n +1} - \beta _{1} U^{n +1} e^{i \overline{I} \theta } \biggr). \end{aligned}$$

Simplification of (26) leads to

27 $$ U^{n +1} - U^{n - 1} = \overline{a} \bigl( \overline{d} \alpha _{1} ( 2 \cos \theta ) U^{n} - 2 \alpha _{1} \overline{d} U^{n +1} - \beta _{1} \Delta t U^{n +1} \bigr), $$

where $\overline{d} = \frac{\Delta t}{( \Delta x )^{2}}$. Equation (27) can be expressed as

28 $$ U^{n +1} = b_{1} U^{n} + b_{2} U^{n - 1}, $$

where $b_{1} = \frac{2 \overline{a} \overline{d} \alpha _{1} \cos \theta }{1+(2 \alpha _{1} \overline{d} + \beta _{1} \Delta t ) \overline{a}}$, $b_{2} = \frac{1}{1+ \overline{a} (2 \alpha _{1} \overline{d} + \beta _{1} \Delta t )}$.

Consider one more equation of the form

29 $$ U^{n} = U^{n} + O U^{n - 1}. $$

The matrix-vector equation can be constructed as

30 $$ \begin{bmatrix} U^{n +1}\\ U^{n} \end{bmatrix} = \begin{bmatrix} b_{1} & b_{2}\\ 1 & 0 \end{bmatrix} \begin{bmatrix} U^{n}\\ U^{n - 1} \end{bmatrix}. $$

For this case, the amplification factor is a matrix, and the condition of stability can be imposed on the eigenvalues of this matrix, which are expressed as

31 $$ \vert \lambda _{1} \vert \leq 1\quad \text{and} \quad \vert \lambda _{2} \vert \leq 1, $$

where $\lambda _{1} = \frac{b_{1}}{2} - \frac{1}{2} \sqrt{b_{1}^{2} +4 b_{2}}$, $\lambda _{2} = \frac{b_{1}}{2} + \frac{1}{2} \sqrt{b_{1}^{2} +4 b_{2}}$.

Let $d = \alpha _{1} \overline{d}$ and $\overline{\beta } = \beta _{1} \Delta t$, then $b_{1}$ and $b_{2}$ can be expressed as

32 $$ b_{1} = \frac{4 d\cos\theta }{1+ \beta +2 d},\qquad b_{2} = - \frac{- 1+ \beta +2 d}{1+ \beta +2 d}. $$

Since the eigenvalues $\lambda _{1}$ and $\lambda _{2}$ contain a fractional power, before finding stability conditions, one can first find the region that corresponds to real eigenvalues. For this reason, the expression $b_{1}^{2} +4 b_{2}$ should be non-negative. So,

33 $$ b_{1}^{2} +4 b_{2} \geq 0. $$

For $\cos \theta =0 $

$$ - \frac{4 ( - 1+\beta +2d )}{1+\beta +2d} \geq 0. $$

This implies $- 1+\beta +2d \leq 0$.

This implies

34 $$ d \leq \frac{1 - \beta }{2}. $$

So real eigenvalues correspond to the region $d \leq \frac{1 - \beta }{2}$ so in this region condition on the eigenvalue $\lambda _{1}$ can be expressed as

$$ - 1 \leq \lambda _{1} \leq 1. $$

Therefore,

35 $$ - 1 \leq \frac{4d\cos\theta }{2 ( 1+\beta +2d )} - \frac{1}{2} \sqrt{ \biggl( \frac{4d\cos\theta }{1+\beta +2d} \biggr)^{2} - \frac{4 ( - 1+\beta +2d )}{1+\beta +2d}}. $$

Since $\vert \cos \theta \vert \leq 1$, consider first $\cos \theta = - 1$ so inequality (35) can be expressed as

$$ 0 \leq 8 \beta ^{2} +8\beta +16\beta d $$

which holds for every value of *β* and *d*.

Consider the second case when $\cos \theta =1$ and inequality $- 1 \leq \lambda _{1}$ yields

$$ - 1 \leq \frac{4d}{2 ( 1+\beta +2d )} - \frac{1}{2} \sqrt{ \biggl( \frac{4d}{1+\beta +2d} \biggr)^{2} - \frac{4 ( - 1+\beta +2d )}{1+\beta +2d}}. $$

This implies

$$ 0 \leq 4 ( \beta +4d )^{2} +4 \beta ^{2} +16d $$

which is also true for every value of *β* and *d*.

Consider the case now when $\lambda _{1} \leq 1$ and $\cos \theta = - 1$,

$$ \frac{- 4d}{2 ( 1+\beta +2d )} - \frac{1}{2} \sqrt{ \biggl( \frac{4d}{1+\beta +2d} \biggr)^{2} - \frac{4 ( - 1+\beta +2d )}{1+\beta +2d}} \leq 1, $$

and hence $- 2 - 2\beta - 8d \leq \sqrt{- 4 \beta ^{2} - 16d+4}$.

This is also true because the negative number is always less than the positive number for $d \leq \frac{1 - \beta }{2}$.

The fourth case, when $\lambda _{1} \leq 1$ and $\cos \theta =1$, yields

$$ - 2 - 2\beta \leq \sqrt{- 4 \beta ^{2} - 16d+4}, $$

This is also true for every *β* and *d*.

Therefore four cases for eigenvalues $\lambda _{1}$ have been discussed, and inequality $\vert \lambda _{1} \vert \leq 1$ holds for every value of *β* and *α* when $d \leq \frac{1 - \beta }{2}$.

Four cases for the second eigenvalue $\vert \lambda _{2} \vert \leq 1$ can be discussed at extreme values of cos*θ*, so four cases are given as when $- 1 \leq \lambda _{2}$ and $\cos \theta = - 1$, and the following inequality can be obtained:

$$ - 2 - 2\beta \leq \sqrt{- 4 \beta ^{2} - 16d+4} $$

when $- 1 \leq \lambda _{2}$ and $\cos \theta =1$, the inequality can be obtained in the form

$$ - 2 - 2\beta - 8d \leq \sqrt{- 4 \beta ^{2} - 16d+4} $$

when $\lambda _{2} \leq 1$ and $\cos \theta = - 1$, an inequality can be expressed in the form of

$$ 0 \leq 4 \beta ^{2} +16\beta d+ ( 2\beta +8d )^{2} +4(2 \beta +8d) $$

and, in the last case when $\lambda _{2} \leq 1$ and $\cos \theta =1$, an inequality can be obtained in the form

$$ 0 \leq 2 \beta ^{2} +2\beta +4 d^{2}. $$

So all inequalities for $- 1 \leq \lambda _{2}$ and $\lambda _{2} \leq 1$ are satisfied with the extreme value of cos*θ*. Thus $\vert \lambda _{1} \vert \leq 1$ and $\vert \lambda _{2} \vert \leq 1$ are true for every *β* and *d* when $d \leq \frac{1 - \beta }{2}$.

The complex eigenvalues are obtained for the region $d \geq \frac{1 - \beta }{2}$. For this case, the stability conditions $\vert \lambda _{1} \vert \leq 1$ and $\vert \lambda _{2} \vert \leq 1$ are expressed as

$$\begin{aligned}& \biggl\vert \frac{b_{1}}{2} - \frac{\overline{I}}{2} \sqrt{- b_{1}^{2} - 4 b_{2}} \biggr\vert ^{2} \leq 1\quad \text{and}\quad \biggl\vert \frac{b_{1}}{2} + \frac{\overline{I}}{2} \sqrt{- b_{1}^{2} - 4 b_{2}} \biggr\vert ^{2} \leq 1, \\& \frac{b_{1}^{2}}{4} + \frac{1}{4} \bigl( - b_{1}^{2} - 4 b_{2} \bigr) \leq 1. \end{aligned}$$

Hence, $- b_{2} \leq 1$ and so

$$ \frac{- 1+\beta +2d}{1+\beta +2d} \leq 1, $$

which is valid for every value of *β* and *d*. Thus the proposed scheme is unconditionally stable for Eq. (23), which is considered for the general type of epidemic disease model or parabolic partial differential equations having some source term(s).

## Consistency of scheme

Taylor series expansions prove the consistency of Eq. (1). For this reason, consider the Taylor series expansions for $S_{i}^{n+1}$ and $S_{i}^{n - 1}$ given in (11) and (12) and the following Taylor series expansions:

36 $$\begin{aligned}& S_{i+1}^{n} = S_{i}^{n} + \Delta x \biggl( \frac{\partial S}{\partial x} \biggr)_{i}^{n} + \frac{( \Delta x)^{2}}{2} \biggl( \frac{\partial ^{2} S}{\partial x^{2}} \biggr)_{i}^{n} +O \bigl( ( \Delta x )^{3} \bigr), \end{aligned}$$

37 $$\begin{aligned}& S_{i - 1}^{n} = S_{i}^{n} - \Delta x \biggl( \frac{\partial S}{\partial x} \biggr)_{i}^{n} + \frac{( \Delta x)^{2}}{2} \biggl( \frac{\partial ^{2} S}{\partial x^{2}} \biggr)_{i}^{n} +O \bigl( ( \Delta x )^{3} \bigr). \end{aligned}$$

So, using expansions (36)–(37), the following equation can be obtained:

38 $$ S_{i+1}^{n} - 2 S_{i}^{n+1} + S_{i - 1}^{n} = ( \Delta x )^{6} \biggl( \frac{\partial ^{2} S}{\partial x^{2}} \biggr)_{i}^{n} - 2 \Delta t \biggl( \frac{\partial S}{\partial x} \biggr)_{i}^{n} +O \bigl( ( \Delta t )^{2}, ( \Delta x )^{4} \bigr). $$

Substituting (11), (12), and (38) into the discretized form (10) yields

39 $$\begin{aligned}& S_{i}^{n} + \Delta t \biggl( \frac{\partial S}{\partial t} \biggr)_{i}^{n} +O \bigl( ( \Delta t )^{2} \bigr) \\& \quad = S_{i}^{n} - \Delta t \biggl( \frac{\partial S}{\partial t} \biggr)_{i}^{n} +O \bigl( ( \Delta t )^{2} \bigr) + \Delta ta \biggl\{ d_{1} \biggl( \frac{\partial ^{2} S}{\partial x^{2}} \biggr)_{i}^{n} - 2 d_{1} \frac{\Delta t}{ ( \Delta x )^{2}} \biggl( \frac{\partial S}{\partial t} \biggr)_{i}^{n} \\& \qquad {}- \alpha A_{i}^{n} S_{i}^{n} - \alpha A_{i}^{n} ( \Delta t ) \biggl( \frac{\partial S}{\partial t} \biggr)_{i}^{n} - \beta I_{i}^{n} S_{i}^{n} - \beta I_{i}^{n} ( \Delta t ) \biggl( \frac{\partial S}{\partial t} \biggr)_{i}^{n} +O \bigl( ( \Delta t )^{2}, ( \Delta x )^{2} \bigr) \biggr\} . \end{aligned}$$

Combining the first derivative in the time terms and simplified Eq. (39) gives

40 $$\begin{aligned}& \bigl( 1 - 2d d_{1} - \Delta t \bigl( \alpha A_{i}^{n} +\beta I_{i}^{n} \bigr) \bigr) \biggl( \frac{\partial S}{\partial t} \biggr)_{i}^{n} \end{aligned}$$

41 $$\begin{aligned}& \quad = d_{1} \biggl( \frac{\partial ^{2} S}{\partial x^{2}} \biggr)_{i}^{n} - \alpha A_{i}^{n} S_{i}^{n} - \beta I_{i}^{n} S_{i}^{n} + \bigl( -2d d_{1} - \alpha \Delta t A_{i}^{n} - \beta \Delta t I_{i}^{n} \bigr) \biggl( \frac{\partial S}{\partial t} \biggr)_{i}^{n} \\& \qquad {} +O \bigl( ( \Delta t )^{2}, ( \Delta x )^{2} \bigr). \end{aligned}$$

Canceling the same terms on both sides of Eq. (41) yields

42 $$ \biggl( \frac{\partial S}{\partial t} \biggr)_{i}^{n} = d_{1} \biggl( \frac{\partial ^{2} S}{\partial x^{2}} \biggr)_{i}^{n} - \alpha A_{i}^{n} S_{i}^{n} - \beta I_{i}^{n} S_{i}^{n} +O \bigl( ( \Delta t )^{2}, ( \Delta x )^{2} \bigr). $$

By incorporating consistency conditions $\Delta t \rightarrow 0$, $\Delta x \rightarrow 0$ in (42) yields the original Eq. (1) evaluated at the *i*th grid point and at time level *n*.

## Convergence of scheme

The stability has been proved for the linear differential equation representing any susceptible, exposed, asymptomatic, infectious, and recovered individuals. The convergence is given for the first two Eqs. (1) and (2), which are merged and form a single linear equation expressed as

43 $$ \frac{\partial E}{\partial t} = d_{2} \frac{\partial ^{2} E}{\partial x^{2}} - \frac{\partial S}{\partial t} + d_{1} \frac{\partial ^{2} S}{\partial x^{2}} - \gamma E. $$

Discretize Eq. (43) using a proposed scheme with unknown parameter in the following manner:

44 $$\begin{aligned}& \frac{E_{i}^{n+1} - E_{i}^{n - 1}}{2 \Delta t} \\& \quad = \check{a} \biggl( d_{2} \frac{E_{i+1}^{n} - 2 E_{i}^{n+1} + E_{i - 1}^{n}}{ ( \Delta x )^{2}} - \frac{S_{i}^{n+1} - S_{i}^{n - 1}}{2 \Delta t} + d_{1} \frac{S_{i+1}^{n} - 2 S_{i}^{n+1} + S_{i - 1}^{n}}{ ( \Delta x )^{2}} - \gamma E_{i}^{n+1} \biggr). \end{aligned}$$

Taylor series expansion for $E_{i}^{n+1}$ and $E_{i}^{n - 1}$ can be incorporated to Eq. (44) giving

45 $$\begin{aligned}& \begin{gathered} E_{i}^{n} + \Delta t \biggl( \frac{\partial E}{\partial t} \biggr)_{i}^{n} = E_{i}^{n} - \Delta t \biggl( \frac{\partial E}{\partial t} \biggr)_{i}^{n} +2 \Delta t \check{a} \begin{pmatrix} ( \frac{\partial E}{\partial t} )_{i}^{n} - 2 \frac{\Delta t}{ ( \Delta x )^{2}} ( \frac{\partial E}{\partial t} )_{i}^{n} -\\ ( \Delta t ) \gamma ( \frac{\partial E}{\partial t} )_{i}^{n} +O ( ( \Delta t )^{2}, ( \Delta x )^{2} ) \end{pmatrix}, \\ 1= \check{a} \bigl( 1 - 2d - ( \Delta t ) \gamma \bigr). \end{gathered} \end{aligned}$$

This implies the expression for the unknown parameter

46 $$ \check{a} = \frac{1}{ ( 1 - 2d - ( \Delta t ) \gamma )}. $$

Thus Eq. (44) is expressed in the form of

47 $$\begin{aligned} E_{i}^{n+1} =& E_{i}^{n - 1} +2 \Delta t \check{a} \biggl\{ d_{2} \frac{E_{i+1}^{n} - 2 E_{i}^{n+1} + E_{i - 1}^{n}}{ ( \Delta x )^{2}} \\ &{} - ( 1+2d d_{1} ) \frac{S_{i}^{n+1} - S_{i}^{n - 1}}{2 \Delta t} + d_{1} \frac{S_{i+1}^{n} - 2 S_{i}^{n+1} + S_{i - 1}^{n}}{ ( \Delta x )^{2}} - \gamma E_{i}^{n+1} \biggr\} . \end{aligned}$$

Let $e_{2,i}^{n+1}$ and $e_{1,i}^{n+1}$ be the difference between the exact solution and numerical solution for exposed and susceptible individuals computed at grid point *i* and at time level *n*. Thus the corresponding error equation for Eq. (47) is expressed as

48 $$ e_{2,i}^{n+1} = e_{2,i}^{n - 1} +2 \Delta t \check{a} \left \{ \textstyle\begin{array}{l} d_{2} \frac{e_{2,i+1}^{n} - 2 e_{2,i}^{n+1} + e_{2,i - 1}^{n}}{ ( \Delta x )^{2}} - ( 1+2d d_{1} ) \frac{e_{1,i}^{n+1} - e_{1,i}^{n - 1}}{2 \Delta t} \\ + d_{1} \frac{e_{1,i+1}^{n} - 2 e_{1,i}^{n+1} + e_{1,i - 1}^{n}}{ ( \Delta x )^{2}} - \gamma e_{2,i}^{n+1} \end{array}\displaystyle \right \} , $$

where *ǎ* is given in Eq. (46).

Combining $e_{2,i}^{n+1}$ on the left side of Eq. (48) gives

49 $$ (1+4d d_{2} \check{a} +2 \Delta t \check{a} \gamma )e_{2,i}^{n+1} = e_{2,i}^{n - 1} +2 \Delta t \check{a} \left \{ \textstyle\begin{array}{l} d_{2} \frac{e_{2,i+1}^{n} + e_{2,i - 1}^{n}}{ ( \Delta x )^{2}} -\\ ( 1+2d d_{1} ) \frac{e_{1,i}^{n+1} - e_{1,i}^{n - 1}}{2 \Delta t} +\\ d_{1} \frac{e_{1,i+1}^{n} - 2 e_{1,i}^{n+1} + e_{1,i - 1}^{n}}{ ( \Delta x )^{2}} - \gamma e_{2,i}^{n+1} \end{array}\displaystyle \right \} . $$

Applying absolute values on both sides of Eq. (49) yields

50 $$\begin{aligned}& \vert 1+4d d_{2} \check{a} +2 \Delta t \check{a} \gamma \vert \bigl\vert e_{2,i}^{n+1} \bigr\vert \\& \quad = \bigl\vert e_{2,i}^{n - 1} \bigr\vert +2 \Delta t \vert \check{a} \vert \biggl\{ d_{2} \frac{ \vert e_{2,i+1}^{n} \vert + \vert e_{2,i - 1}^{n} \vert }{ ( \Delta x )^{2}} - \vert 1+2d d_{1} \vert \frac{ \vert e_{1,i}^{n+1} \vert - \vert e_{1,i}^{n - 1} \vert }{2 \Delta t} \\& \qquad {}+ \vert d_{1} \vert \frac{ \vert e_{1,i+1}^{n} \vert - 2 \vert e_{1,i}^{n+1} \vert + \vert e_{1,i - 1}^{n} \vert }{ ( \Delta x )^{2}} \biggr\} . \end{aligned}$$

Let $e^{n} = \max \{ \vert e_{1,i}^{n} \vert ,\ \vert e_{2,i}^{n} \vert \} $, then the inequality (50) can be expressed as

51 $$\begin{aligned}& \vert 1+4d d_{2} \check{a} +2 \Delta t \check{a} \gamma \vert - 2 \Delta t \vert \check{a} \vert \biggl( \frac{ \vert 1+2d d_{1} \vert }{2 \Delta t} + \frac{2 \vert d_{1} \vert }{ ( \Delta x )^{2}} \biggr) e^{n+1} \\& \quad \leq e^{n - 1} +2 \Delta t \vert \check{a} \vert \biggl\{ \vert d_{2} \vert \frac{e^{n}}{ ( \Delta x )^{2}} + \vert 1+2d d_{1} \vert \frac{e^{n - 1}}{2 \Delta t} + \vert d_{1} \vert \frac{2 e^{n}}{ ( \Delta x )^{2}} \biggr\} . \end{aligned}$$

Equation (51) can be expressed as

52 $$ \delta e^{n+1} \leq \delta _{1} e^{n - 1} + \delta _{2} e^{n} +CO \bigl( ( \Delta t )^{2}, ( \Delta x )^{2} \bigr), $$

where $\delta = \vert 1+4d d_{2} \check{a} +2 \Delta t \check{a} \gamma \vert - 2\ \vert \check{a} \vert ( \frac{ \vert 1+2d d_{1} \vert d}{2} + \frac{2d \vert d_{1} \vert }{ ( \Delta x )^{2}} )$, $\delta _{1} =1+ \vert \check{a} \vert \vert 1+2d d_{1} \vert $ and $\delta _{2} =2 \vert \check{a} \vert \vert d_{2} \vert d+4 \vert \check{a} \vert \vert d_{1} \vert d$.

Let $\delta >0$, then inequality (52) yields

53 $$ e^{n+1} \leq \frac{\delta _{1}}{\delta } e^{n - 1} + \frac{\delta _{2}}{\delta } e^{n} + \frac{1}{\delta } CO \bigl( ( \Delta t )^{2}, ( \Delta x )^{2} \bigr). $$

Let $e^{n - 1} = \max ( e^{n - 1},\ e^{n} )$ and $\frac{\delta _{1} + \delta _{2}}{\delta } = \delta _{3}$, then Eq. (53) is given by

54 $$ e^{n+1} \leq \delta _{3} e^{n - 1} + C_{1} O \bigl( ( \Delta t )^{2}, ( \Delta x )^{2} \bigr), $$

where $C_{1}$ is the coefficient of the leading remainder term.

For $n=1$ in Eq. (54)

55 $$ e^{2} \leq \delta _{3} e^{0} + C_{1} O \bigl( ( \Delta t )^{2}, ( \Delta x )^{2} \bigr). $$

Since $e^{0} =0$ due to the initial condition, Eq. (55) can be expressed as

56 $$ e^{2} \leq C_{1} O \bigl( ( \Delta t )^{2}, ( \Delta x )^{2} \bigr). $$

For $n=2$ in Eq. (54)

57 $$ e^{3} \leq \delta _{3} e^{1} + C_{1} O \bigl( ( \Delta t )^{2}, ( \Delta x )^{2} \bigr). $$

Let the error at the first time obtained be $e^{1} \leq M$ then (57) is expressed as

58 $$ e^{3} \leq \delta _{3} M+ C_{1} O \bigl( ( \Delta t )^{2}, ( \Delta x )^{2} \bigr). $$

For $n=3$ in Eq. (54)

59 $$ e^{4} \leq \delta _{3} e^{2} + C_{1} O \bigl( ( \Delta t )^{2}, ( \Delta x )^{2} \bigr) \leq ( \delta _{3} +1) C_{1} O \bigl( ( \Delta t )^{2}, ( \Delta x )^{2} \bigr). $$

For $n=4$ in Eq. (54)

60 $$ e^{5} \leq \delta _{3} e^{3} + C_{1} O \bigl( ( \Delta t )^{2}, ( \Delta x )^{2} \bigr) \leq \delta _{3}^{2} M+ \delta _{3} C_{1} O \bigl( ( \Delta t )^{2}, ( \Delta x )^{2} \bigr). $$

For $n=5$ in Eq. (54)

61 $$ e^{6} \leq \delta _{3} e^{4} + C_{1} O \bigl( ( \Delta t )^{2}, ( \Delta x )^{2} \bigr) \leq \bigl(\delta _{3}^{2} + \delta _{3} +1\bigr)+ C_{1} O \bigl( ( \Delta t )^{2}, ( \Delta x )^{2} \bigr). $$

For $n=6$ in Eq. (54)

62 $$ e^{7} \leq \delta _{3} e^{5} + C_{1} O \bigl( ( \Delta t )^{2}, ( \Delta x )^{2} \bigr) \leq \delta _{3}^{3} M+ \bigl( \delta _{3}^{2} + \delta _{3} +1\bigr)+ C_{1} O \bigl( ( \Delta t )^{2}, ( \Delta x )^{2} \bigr). $$

If this is continued for a finite number of *n* then for even *n*

63 $$\begin{aligned} e^{2n}& \leq \delta _{3}^{n} M+ (\delta _{3}^{n - 1} + \bigl(\delta _{3}^{n - 2} + \cdots +1\bigr)+ C_{1} O \bigl( ( \Delta t )^{2}, ( \Delta x )^{2} \bigr) \\ &= \delta _{3}^{n} M+ \frac{1 ( 1 - \delta _{3}^{n} )}{1 - \delta _{3}} + C_{1} O \bigl( ( \Delta t )^{2}, ( \Delta x )^{2} \bigr). \end{aligned}$$

Equation (63) is obtained by considering even exponent terms.

For odd *n*

64 $$ e^{2n - 1} \leq \bigl(\delta _{3}^{n - 1} + \cdots +1\bigr)+ C_{1} O \bigl( ( \Delta t )^{2}, ( \Delta x )^{2} \bigr) = \frac{ ( 1 - \delta _{3}^{n} )}{1 - \delta _{3}} + C_{1} O \bigl( ( \Delta t )^{2}, ( \Delta x )^{2} \bigr). $$

For large *n* the series $1+ \delta _{3} + \cdots + \delta _{3}^{n - 1}$ will converge if $\vert \delta _{3} \vert \leq 1$.

Similarly, convergence can be found for the cases when $\max ( e^{n - 1}, e^{n} ) = e^{n}$.

This gives convergence of the proposed scheme for the first two Eqs. (1) and (2). Similarly, convergence can be found for the remaining Eqs. (3)–(5).

## Application

The proposed numerical scheme has been constructed and employed for solving the diffusive epidemic model. Initially, the model comprised ordinary differential equations constructed in [6], but it is modified with diffusion effects in this contribution. Due to the fact that diffusion is dependent on a spatial variable, it also makes use of information to transmit individuals from one location to another. The ODEs model [6] only uses the information in time variable, but here time and space both have been utilized, and a diffusive epidemic model has been presented. The main concern is the numerical scheme. Among existing numerical schemes, the present attempt is made to construct a numerical scheme that provides an approximately unconditionally stable solution and explicit in nature. In the literature Du- Fort Frankel’s method exists, which is first-order accurate, explicit, and unconditionally stable but it does not provide conditions to get positive solutions of epidemic models, but the present approach gives the positivity conditions, the conditions on which one can obtain the positive solution. Although the conditions may depend upon the individuals of other categories, it can give some estimate to get a positive solution. The other advantage of this scheme is first-order accuracy for solving partial differential equations. Nonstandard finite difference method is not even first-order accurate, so it may produce some doubt full results but present strategy of constructing scheme is based on applying Taylor series, so theoretically it is first-order accurate which has the advantage for consumption of less time than one of the nonstandard schemes and this can be seen by drawing the graphs on the spatial variable when solving partial differential equations.

Since the von Neumann type boundary conditions are employed on the boundary, it makes the proposed explicit scheme implicit. An additional iterative approach of the Gauss-Seidel iterative method is also employed to solve the resulting difference equations. The iterative approach tackles the von Neumann type boundary condition on the left boundary. The von Neumann type boundary condition can be incorporated explicitly if it is employed on the last grid point. In this case, the backward difference formula can be considered to find each dependent variable’s value on the last grid point. But, for the first gird point, the first-order forward difference formula using Gauss-Seidel iterative method and this can be expressed in the following manner:

65 $$ \frac{S_{i+1}^{n+1,k} - S_{i}^{n+1,k+1}}{\Delta t} =0, $$

which implies

66 $$ S_{i}^{n+1,k+1} = S_{i+1}^{n+1,k}. $$

Similarly, this formula can be employed on all boundary conditions imposed on exposed, asymptomatic, infected and recovered individuals at the left endpoint. The boundary condition on the right endpoint can be tackled explicitly. Using the Gauss-Seidel iterative method, it reads

67 $$ S_{i}^{n+1,k+1} = S_{i-1}^{n+1,k+1}. $$

When the Gauss-Seidel iterative is employed on the difference equations obtained by discretizing Eq. (1) using the proposed scheme, it reads

68 $$ S_{i}^{n +1,k+1} = S_{i}^{n - 1,k+1} + \Delta t a_{1} \left \{ \textstyle\begin{array}{l} d_{1} \frac{S_{i +1}^{n,k+1} - 2 S_{i}^{n +1,k+1} + S_{i - 1}^{n,k+1}}{ ( \Delta x )^{2}}\\ - \alpha A_{i}^{n,k+1} S_{i}^{n +1,k+1} - \beta I_{i}^{n,k+1} S_{i}^{n +1,k+1} \end{array}\displaystyle \right \} , $$

where $a_{1} =a$ is given in (16).

**Figure 1:**
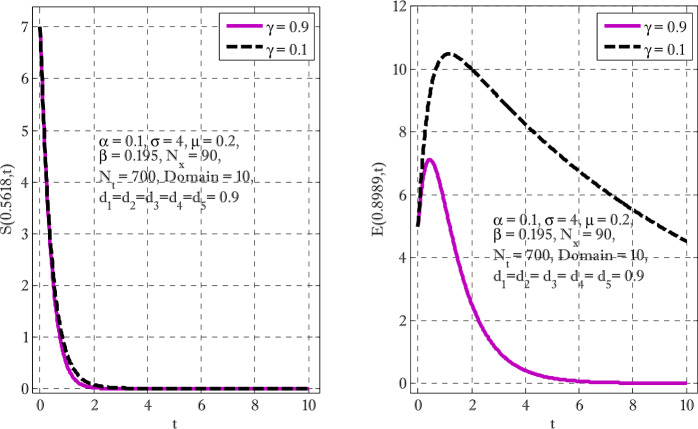
Susceptible and exposed individuals with the variation of conversion rate *γ*

**Figure 2:**
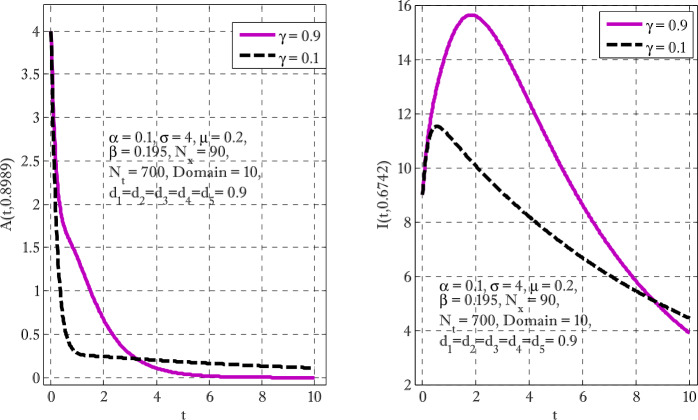
Asymptomatic and infected individuals with the variation of conversion rate *γ*

**Figure 3:**
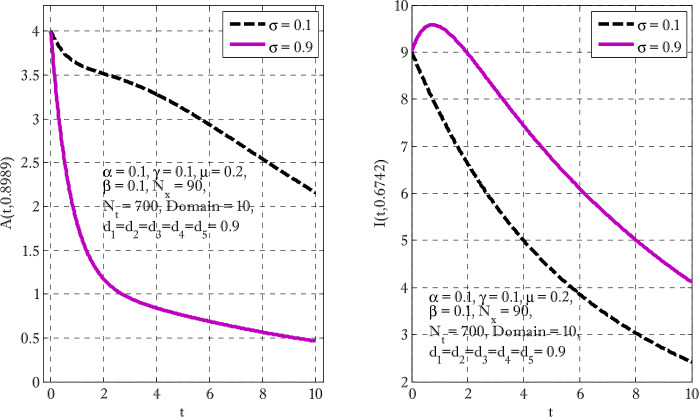
Asymptomatic and infected individuals with the variation of growth rate parameter *σ*

**Figure 4:**
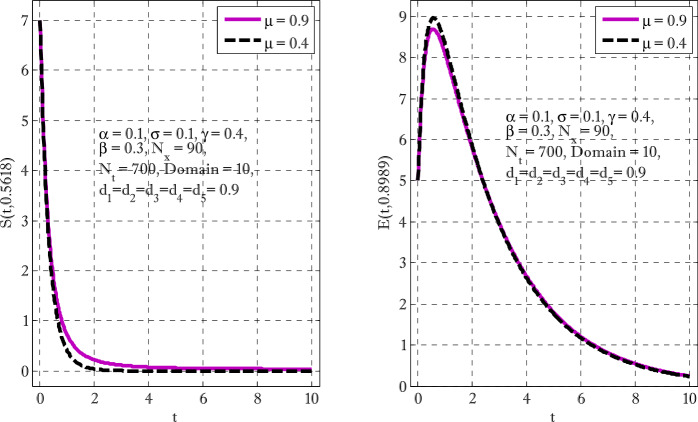
Susceptible and exposed individuals with the variation of *μ*

**Figure 5:**
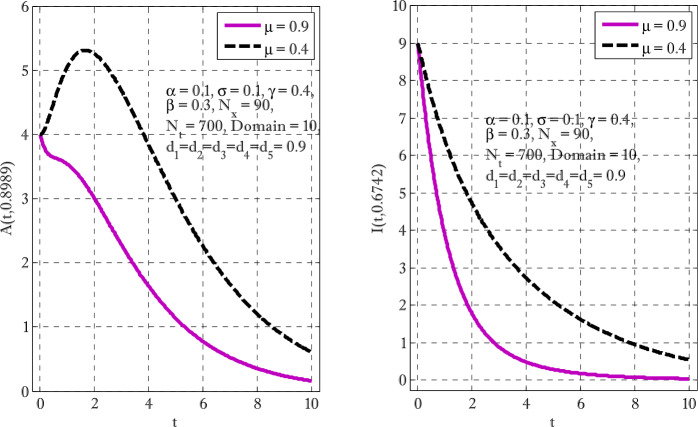
Asymptomatic and infected individuals with the variation of recovery rate parameter *μ*

**Figure 6:**
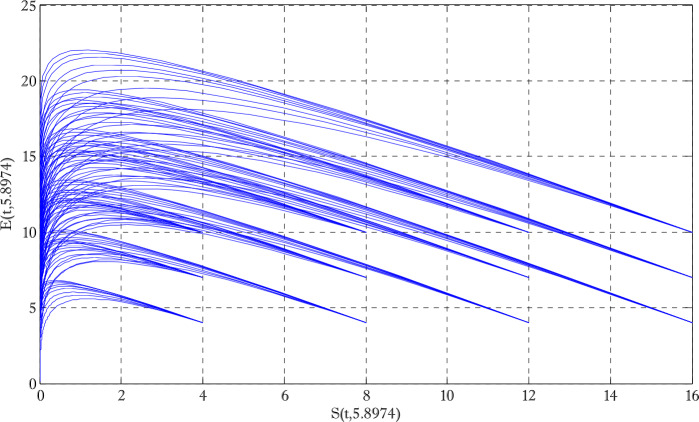
Two dimensional phase portrait of susceptible and exposed individuals

**Figure 7:**
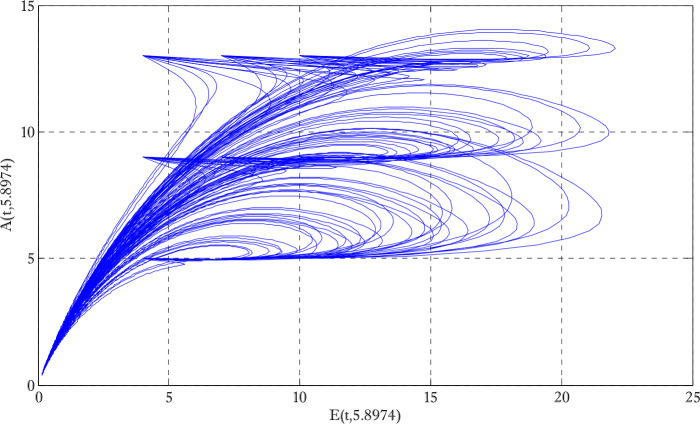
Two dimensional phase portrait of asymptomatic and exposed individuals

**Figure 8:**
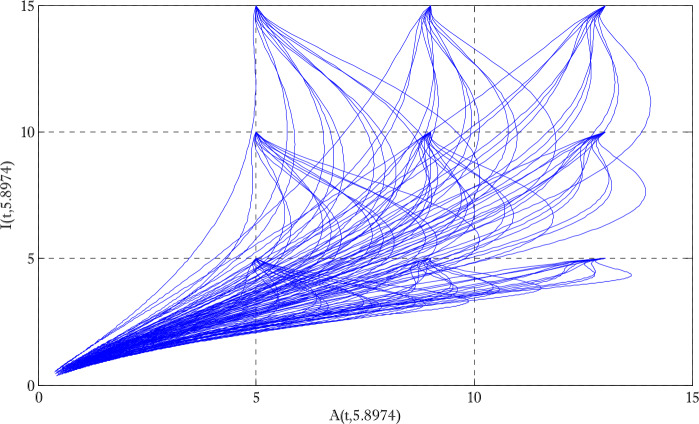
Two dimensional phase portrait of infected and asymptomatic individuals

**Figure 9:**
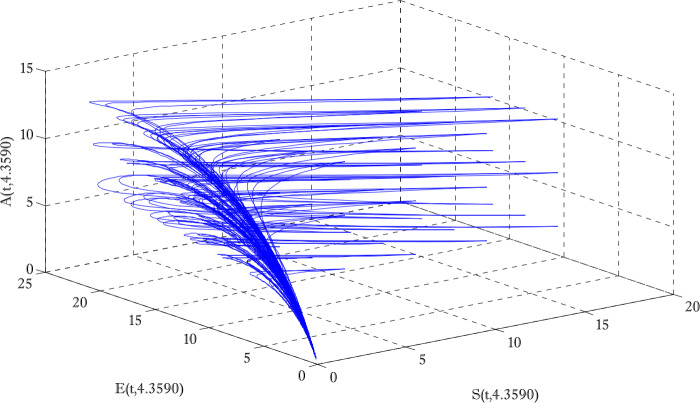
Three dimensional phase portrait of susceptible, exposed and asymptomatic individuals

**Figure 10:**
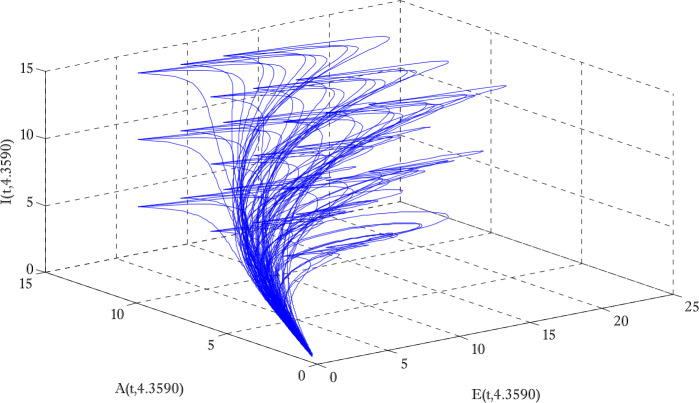
Three dimensional phase portrait of exposed, asymptomatic and infected individuals

**Figure 11:**
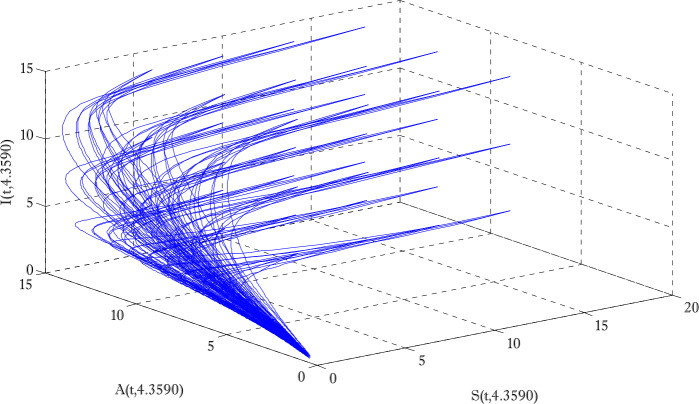
Three dimensional phase portrait of susceptible, asymptomatic and infected individuals

Figure 1 shows the behaviors of susceptible and exposed individuals over time when the rate *γ* varies. Figure 1 also indicates that both susceptible and exposed individuals are decreasing with an increase in the conversion rate *γ*. The exposed people will decrease due to their transmission from the exposed category to the asymptomatic category, and since both asymptomatic and infected individuals have to increase. Decreasing behaviors so susceptible people decrease mostly, but these people also have increasing behavior which is very small and can only be seen on a very small scale. Figure 2 shows the asymptomatic and infected individuals over time. Both categories have increasing and decreasing behavior due to the increase in conversion rate *γ*. Figure 3 presents asymptomatic and infected individuals over time. Figure 3 clearly shows the enhancement and decay of infected and asymptomatic people by enhancing the growth rate *σ* because asymptomatic people will shift the category of asymptomatic individuals to infected individuals. Figure 4 shows the behavior of susceptible and exposed individuals. Figure 4 shows that susceptible people are increasing and exposed people are decreasing by enhancing the recovery rate parameter *μ*. Figure 5 shows the behavior of asymptomatic and infected individuals over time. It is seen clearly from Fig. 5 that both categories of individuals decrease by increasing the recovery rate parameter *μ*. Figures 6-11 show the phase portraits in two and three dimensions. These phase portraits show the relationships between different individuals in the modified diffusive COVID-19 epidemic model. The set of initial conditions to draw these figures are given as

$$ a\epsilon \{ 4,8,12,16 \} ,\qquad b\epsilon \{ 4,7,10 \} ,\qquad c\epsilon \{ 5,9,13 \} \quad \text{and}\quad c_{1} \epsilon \{ 5,10,15 \}, $$

where $a=S ( 0,x )$, $b=E ( 0,x )$, $c=A ( 0,x )$ and $c_{1} =I ( 0,x )$. The values of the parameters are given as

$$\begin{aligned}& \alpha =0.1,\qquad \beta =0.3, \qquad \gamma =0.4, \qquad \sigma =0.1, \qquad \mu =0.4, \\& d_{1} = d_{2}= d_{3} = d_{4} = d_{5} =0.9,\qquad N_{x} =40,\qquad N_{t} =190. \end{aligned}$$

**Figure 12:**
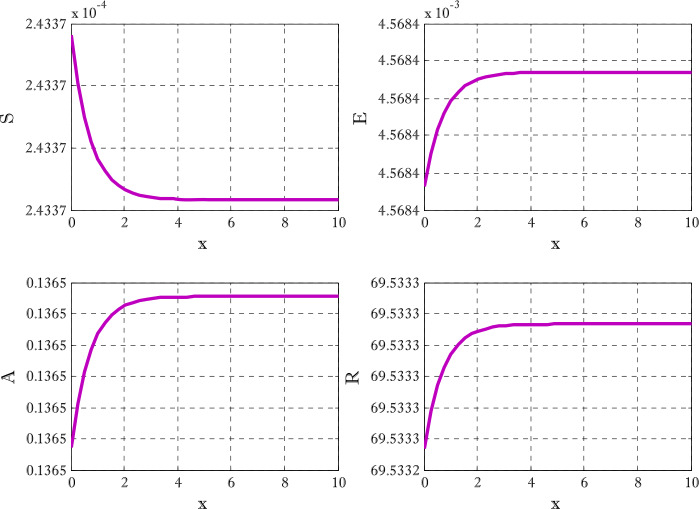
Solution obtained by Euler method using $t=10$, $N_{x} =40$, $N_{t} =190$, $\alpha =0.1$, $\beta =0.3$, $\gamma =0.4$, $\sigma =0.1$, $\mu =0.4$, $d_{1} = d_{2} =d_{3} = d_{3} = d_{5} =0.9$

**Figure 13:**
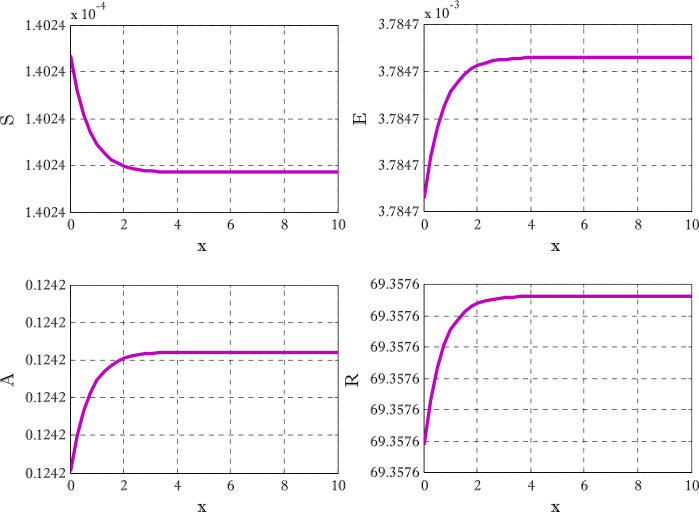
Solution obtained by proposed scheme $t=10$, $N_{x} =40$, $N_{t} =290$, $\alpha =0.1$, $\beta =0.3$, $\gamma =0.4$, $\sigma =0.1$, $\mu =0.4$, $d_{1} = d_{2} =d_{3} = d_{3} = d_{5} =0.9$, $a=-0.3$, $b=1.3$

**Figure 14:**
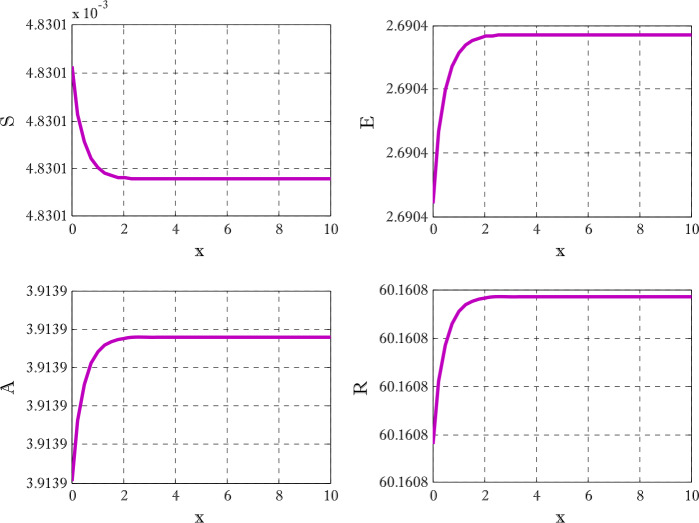
Solution obtained by proposed scheme $t=10$, $N_{x} =40$, $N_{t} =190$, $\alpha =0.1$, $\beta =0.3$, $\gamma =0.4$, $\sigma =0.1$, $\mu =0.4$, $d_{1} = d_{2} =d_{3} = d_{3} = d_{5} =0.9$

**Figure 15:**
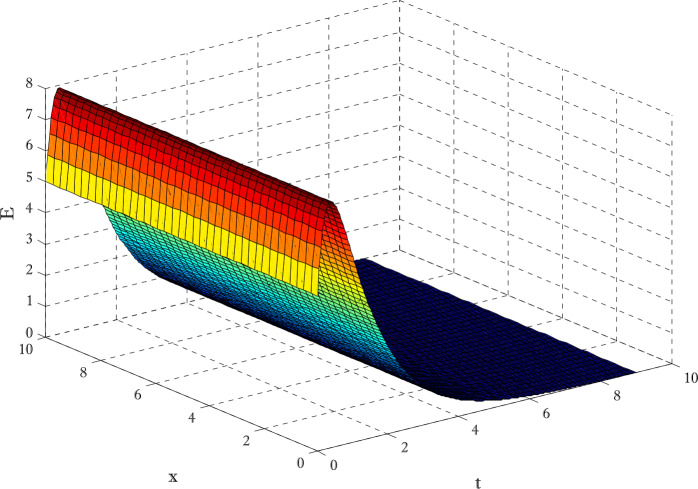
Surface plot for exposed individuals $t=9$, $N_{x} =40$, $N_{t} =190$, $\alpha =0.1$, $\beta =0.3$, $\gamma =0.4$, $\sigma =0.1$, $\mu =0.4$, $d_{1} = d_{2} =d_{3} = d_{3} = d_{5} =0.9$, $a=-0.3$, $b=1.3$

**Figure 16:**
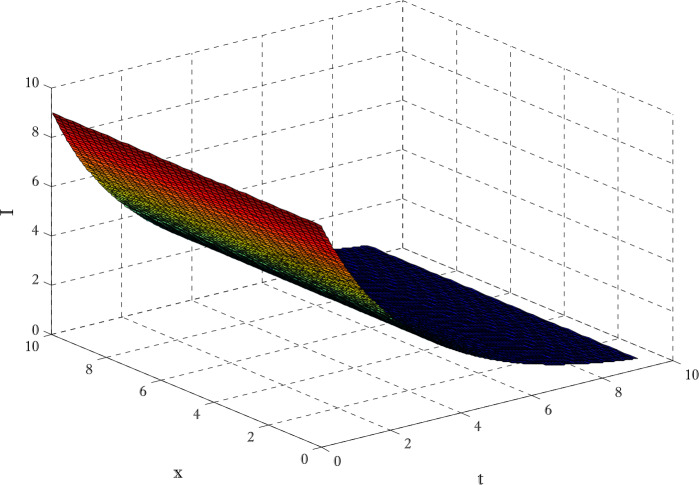
Surface plot for infective individuals $t=9$, $N_{x} =40$, $N_{t} =190$, $\alpha =0.1$, $\beta =0.3$, $\gamma =0.4$, $\sigma =0.1$, $\mu =0.4$, $d_{1} = d_{2} =d_{3} = d_{3} = d_{5} =0.9$, $a=-0.3$, $b=1.3$

Figures 12-14 are drawn to elucidate the comparison of three schemes. The solution obtained by the proposed scheme and the nonstandard finite difference method has been displayed in Figs. 13 and 14, respectively. The values on the *y*-axis can be seen for clear comparison between three scheme. The proposed scheme produced the solution near to first-order method but nonstandard finite difference method produced the solution a little away from the solution obtained by first-order method (forward Euler method). These figures show the advantage of using proposed scheme. Since the Euler method does not converge on those time levels which are used by proposed scheme so larger number of time levels $( N_{t} )$ are used to get converged solution. Figures 15 and 16 are surface plots for exposed and infective individuals. These figures are three dimensional views of exposed and infective individuals over *t* and *x*-axis. From Figs. 15 and 16, it can be observed how plotted individuals behave on *t*- and *x*-axis. In the captions of these figures $N_{x}$ and $N_{t}$ denote the number of grid points and number of time levels, respectively. The parameter *a* and *b* are used for choosing a particular scheme.

## Conclusion

A first-order in time and second-order in explicit space scheme has been proposed. The scheme is unconditionally stable according to von Neumann’s stability criteria. The proposed scheme provided conditions to obtain a positive solution. The solution’s positivity was dependent upon the step sizes in time and space and the chosen values of the parameters contained in the considered diffusive COVID-19 model. The consistency of the scheme has been proved, and convergence conditions have also been found. The scheme can be considered to solve related epidemic models and other parabolic partial differential equations.

## Data Availability

The manuscript included all required data and implementing information.
